# Crack Texture Feature Identification of Fiber Reinforced Concrete Based on Deep Learning

**DOI:** 10.3390/ma15113940

**Published:** 2022-06-01

**Authors:** Shuangxi Zhou, Yuan Pan, Xiaosheng Huang, Dan Yang, Yang Ding, Runtao Duan

**Affiliations:** 1 School of Civil Engineering and Architecture, East China Jiao Tong University, Nanchang 330013, China; green.55@163.com (S.Z.); yuanpan227@126.com (Y.P.); gryangdan@163.com (D.Y.); 2 School of Software, East China Jiao Tong University, Nanchang 330013, China; 0126@ecjtu.edu.cn (X.H.); 2020218085400032@ecjtu.edu.cn (R.D.); 3 Department of Civil Engineering, Zhejiang University, Hangzhou 310058, China; 4 Department of Civil Engineering, Zhejiang University City College, Hangzhou 310015, China

**Keywords:** texture features, deep learning, crack identification, concrete

## Abstract

Structural cracks in concrete have a significant influence on structural safety, so it is necessary to detect and monitor concrete cracks. Deep learning is a powerful tool for detecting cracks in concrete structures. However, it requires a large quantity of training samples and is costly in terms of computational time. In order to solve these difficulties, a deep learning target detection framework combining texture features with concrete crack data is proposed. Texture features and pre-processed concrete data are merged to increase the number of feature channels in order to reduce the demand of training samples for the model and improve training speed. With this framework, concrete crack detection can be realized even with a limited number of samples. To accomplish this aim, self-made steel fiber reinforced concrete crack data is used for comparison between our framework and those without texture feature mergence or pre-processed concrete data. The experimental results show that the number of parameters that need to be fitted in the model training and training time can be correspondingly reduced and the detection accuracy can also be improved.

## 1. Introduction

Concrete is one of the most widely used materials in civil engineering. However, concrete is prone to physical and chemical reactions in the external environment, inevitably leading to cracks in concrete. Cracks seriously affect the safety and durability of concrete structures. Therefore, it is of great significance to detect and monitor the health of concrete structures. At present, the concrete crack detection methods mainly include the radar method [1], moiré method [2], infrared thermography method [3], acoustic emission method [4], and holographic interference method [5]. However, these methods are mainly based on contact or embedded methods to detect cracks, and are greatly affected by the external environment such as temperature and humidity. They have limitations in terms of the reliability [6], human experience, and time consumption.

Computer image recognition methods can be used for concrete crack identification. Classical machine vision algorithms mainly include Linear Regression [7], decision tree [8], support vector machine [9], and naive Bayes [10]. Li et al. [11] extracted multi-layer feature segmentation cracks from a complete convolution network and naive Bayes data fusion model. Sharma et al. [12] combined support vector machine and convolutional neural network to identify reinforced concrete cracks. This method has higher recognition accuracy than using convolutional neural networks alone. Prateek et al. [13] used machine vision method to extract image features from the obtained concrete crack images and then trained the system to extract crack information. Choudhary et al. [14] constructed an artificial neural network with fuzzy logic to identify reinforced concrete cracks, and the identification result was ideal. Xu et al. [15] considered the factors of image gray level and pixel rate to identify concrete cracks, and the recognition success rate of cracks larger than 0.3 mm in the image reached 94%. However, traditional algorithms mainly identify the spatial location information, gray value, saturation of images, and cannot extract the deep features of images, which leads to low identification accuracy.

In recent years, the deep learning method has been widely used to identify reinforced concrete cracks [16,17]. Using a deep learning approach to identify cracks and detect targets and segment examples can achieve much better results. Cha et al. [18] constructed a deep convolution network to identify 40,000 concrete crack images, and the accuracy of crack identification was improved compared with that of traditional detection. Gibb et al. [19] proposed adding a genetic algorithm to the deep learning network model to optimize and control the structure and calculation parameters of neural networks. After several iterations of the genetic algorithm, the network model improves the detection accuracy of concrete cracks. Liu et al. [20] constructed a U-net network model to extract the gray scale and spatial information of concrete data for target detection. Compared with the detection accuracy of deep convolution neural network model, the former has better detection results. Dorafshan et al. [21] used the edge detection method and depth convolution network model to detect concrete cracks respectively. The crack detection accuracy of edge detection method was 53–79%, while that of depth convolution network model reached 86%. Zhang et al. [22] put forward a concrete crack detection method using an integrated one-dimensional convolutional neural network (1D-CNN) and long-short memory (LSTM) method in the image frequency domain. The crack detection obtained by this method has high accuracy and is expected to realize real-time detection. Xu et al. [23] used a Faster R-CNN and Mask R-CNN model combined joint training strategy method to detect road cracks. Both models can complete the detection task with only 130+ images used to train each model. Ding et al. [24] proposed an improved Mask R-CNN concrete crack identification model, which had a higher accuracy compared with basic Mask R-CNN. An, Q. [25] constructed a UHK-net network fusing fractal dimension to conduct the semantic recognition of concrete cracks.

Generally, in order to use a deep learning model for training to obtain relevant weights, massive input data (tens or even hundreds of thousands of data points) is required. Multi-level features are extracted from the input data, and the features are used for target detection. However, it is not realistic to obtain a massive quantity of concrete crack data, and it is time-consuming to train the model. Therefore, it is particularly important to find a deep learning method that only needs a small amount of input data to achieve effective detection results.

The image itself contains rich texture features. The texture features of the image describe the arrangement rules of the image and reflect the gray scale variation law [26,27]. Extracting the texture features and spatial correlation features of the original image, and combining these features for visual analysis, allow for excellent performance in many fields such as the biomedical science [28,29,30], industrial automation [31], remote sensing image processing [32,33], and face recognition [34]. By fusing one-dimensional or three-dimensional crack data with the crack features extracted through feature extraction, the dimension of image data is increased and richer crack information is obtained, which makes it possible to reduce the input data.

A deep learning target detection framework is proposed in this work, which combines texture features with concrete crack data. The traditional data and texture features are fused, and the deep learning target detection model is used to mine the depth features of data sources, so as to realize steel fiber reinforced concrete crack detection and case segmentation. Compared with the existing deep learning methods, it can not only reduce the number of parameters that need to be fitted in deep network model training, but also reduce the time consumption of the deep learning model. When there is less model training data, it can obtain better detection results and improve detection accuracy.

## 2. Materials and Methods

### 2.1. Method Introduction

**Figure 1:**
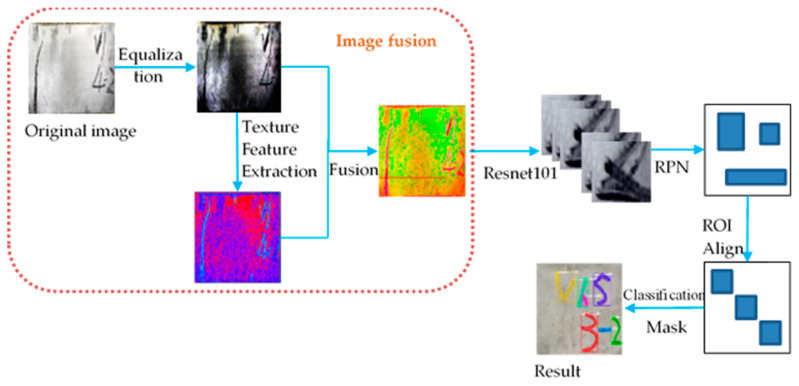
Flowchart of the proposed method for crack detection of concrete images.

The scheme of the fusion target detection framework proposed in this paper is shown in Figure 1. The crack detection method based on texture feature fusion (T-R-CNN) can be divided into five steps: (1) The original steel fiber concrete crack data are processed by histogram equalization and noise elimination. (2) Texture features are extracted from the processed data. (3) The extracted texture feature data is fused with histogram equalization data. (4) A deep learning framework is constructed, which is used to carry out deep learning on the fusion data obtained in Step (3) and extract richer feature layers. (5) After obtaining the characteristic layer, target detection is carried out on the steel fiber concrete data. While extracting texture features in step (2), it is necessary to select traditional texture features according to the actual situation of the data set used in the experiment, and then input the deep learning model.

### 2.2. Principle of Texture Feature Extraction

Texture features, namely gray-level co-occurrence matrix (GLCM), were proposed by Haralick [35]. The relationship between pixels in an image is measured first. Then it is used to precisely reflect image texture roughness, spatial complexity, and repetition direction [36]. It shows that among all the statistical features, entropy and contrast are the two most distinct texture features, while the angular second moment (ASM) and homogeneity reflect the image thickness and local similarity, respectively [37]. Therefore, better target features can be obtained by combining the above four texture features.

Entropy: The internal confusion degree of image pixels, i.e., the randomness of statistical texture distribution. When the pixel values in GLCM show greater randomness, the larger the value of entropy and the more complex the image.

(1) $$t_{1}=-\sum_{i}\sum_{ij}C_{ij}\log C_{ij}$$

Contrast: The gray level variation of image pixels. The greater the contrast, the clearer the image.

(2) $$t_{2}={\sum_{i}\sum_{j}\left(i-j\right)}^{2}C_{ij}$$

Angular second moment: The uniformity and fineness of image gray distribution.

(3) $$t_{3}=\sum_{i}\sum_{j}C_{ij}^{2}$$

Homogeneity: The similarity of image texture. The more similar the local image, the higher the homogeneity value.

(4) $$t_{4}=\sum_{i}\sum_{j}\frac{C_{ij}}{1+{\left(i-j\right)}^{2}}$$

For a color image with a size of M × N, there are three bands, R, G, and B. Four texture features are extracted from these three bands respectively, and the texture features are combined into a three-dimensional matrix P1 of M × N × 12. The enhanced image is a two-dimensional matrix P2 with a size of M × N × 3. The texture feature image and the enhanced image are fused to form a three-dimensional matrix P of M × N × 15, as shown in Equation (5).

P = [P1, P2] (5)

### 2.3. Construction of Deep Learning Framework Based on Texture Features

The deep learning module of T-R-CNN method described includes the input layer, feature extraction layer (residual network Resnet101, feature pyramid network FPN), full connection layer, and loss function. The main structural layers of deep network are described in detail below.

#### 2.3.1. Input Layer

The network input layer in this paper was used to input texture feature data and original data after fusion.

#### 2.3.2. Feature Extraction Layer

The main function of feature extraction layer is to extract deep features from the input layer. Resnet101 residual network model and FPN feature pyramid network are used to fuse feature images to extract deep features.

Deep network usually leads to model over-fitting, gradient disappearance, or gradient explosion while deep residual module can better solve this problem. By adding residual units to the network and increasing the identity mapping connection, the network is at least no worse than the shallow network. Meanwhile, the difference generated by the residual part has a greater impact on the weight, thus ensuring that the gradient value of the back propagation is larger. With the deepening of the network, the network will not degrade, and the depth features can be well extracted.

#### 2.3.3. Full Connection Layer

The main function of the full connection layer is to map the acquired deep features to the training sample labels. At the end of the full connection layer, softmax activation function is loaded to normalize the output value.

#### 2.3.4. Loss Function

The loss function represents the quality of the model. The deep learning model used in this paper was used to achieve classification, target positioning, and semantic segmentation. Therefore, the loss function is composed of the sum of these three loss functions, as shown in Equations (6)–(9)

(6) $$L=L_{cls}+L_{box}+L_{mask}$$

(7) $$L_{cls}=\frac{1}{N_{cls}}\sum_{i}L_{cls}(p_{i},p_{i}*)$$

(8) $$L_{box}(t_{i},t_{i}*)=R(t_{i}-t_{i}*)$$

(9) $$L_{mask}=-\frac{1}{s}\sum_{i}\left[s_{i}*\mathrm{lg}p(s_{i})-(1-s_{i}*)\mathrm{lg}(1-p(s_{i}))\right]$$

where $L_{cls}$ is classification loss function and $p_{i}$ is the probability of predicting the target. $p_{i}*$ indicates whether it is a real target; if it is unity, it is a real target, otherwise it is 0. $L_{box}$ is regression loss function, $t_{i}$ is the parameterized coordinate of the predicted bounding box, and $t_{i}*$ is the bounding box coordinate associated with the anchor point. $L_{mask}$ is semantic segmentation loss function, s is the sum of the total number of a class for each pixel, $s_{i}*$ is the label of the class in which the pixel is located, and $p(s_{i})$ is the probability of predicting the class.

## 3. Results

**Figure 2:**
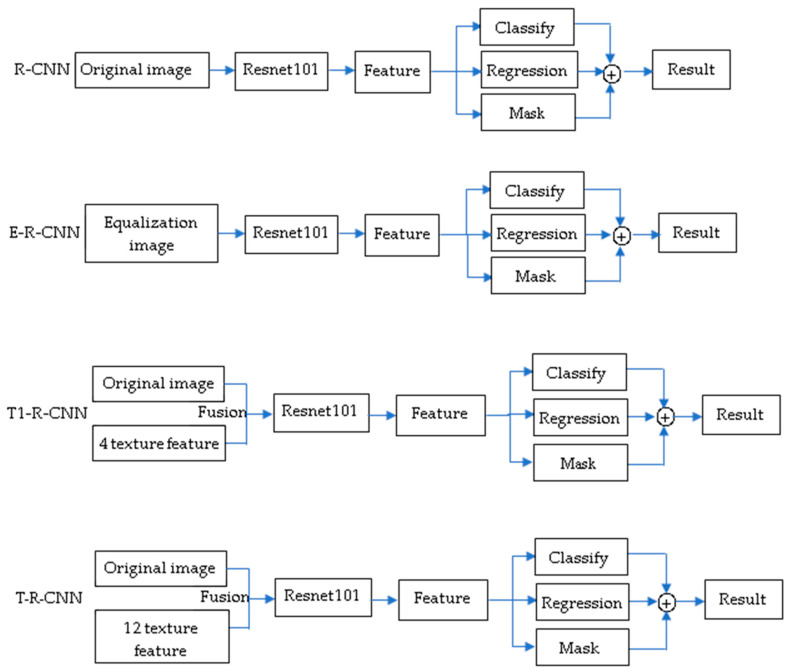
Comparison method.

The experimental purposes of the fusion detection framework proposed in this paper include evaluating the effectiveness and efficiency of the fusion detection framework when the number of training samples is small. In order to fully verify the effectiveness of this method, the proposed T-R-CNN was compared with the other three methods. These methods can be divided into target detection with no fused texture features and target detection with fused texture features. The target detection with no fused texture features includes the detection method based on original data (R-CNN) and the detection method based on preprocessed data (E-R-CNN). The fusion texture feature target detection method (T1-R-CNN) fuses four texture features extracted from R band data. The specific process is shown in Figure 2.

### 3.1. Sample Preparation

#### 3.1.1. Preparation of Steel Fiber Reinforced Concrete

In order to obtain complete concrete crack data, the concrete crack data used in this experiment were self-made steel fiber reinforced concrete test block. Because the process of making concrete test blocks is cumbersome and the cycle is long, 116 prismatic test blocks with the size of 100 × 100 × 400 mm^3^ were made. The experimental raw materials were PO42.5 ordinary cement, medium sand, 5–20 mm gravel, flat copper plated steel fiber, water reducer, etc. In this experiment, firstly, cement and sand were added to dry stirred for 1–2 min. After stirring homogeneously, 85% and 15%water were sequentially added. In order to avoid fiber polymerization in one place, steel fibers were evenly sprinkled and fully stirred. After vibrating the specimens, they were placed indoors for 24 h, then demolded and soaked in water for curing. The specimens were kept fully immersed in water during curing. The curing time of the specimen was 90 days.

#### 3.1.2. Concrete Crack Acquisition

**Figure 3:**
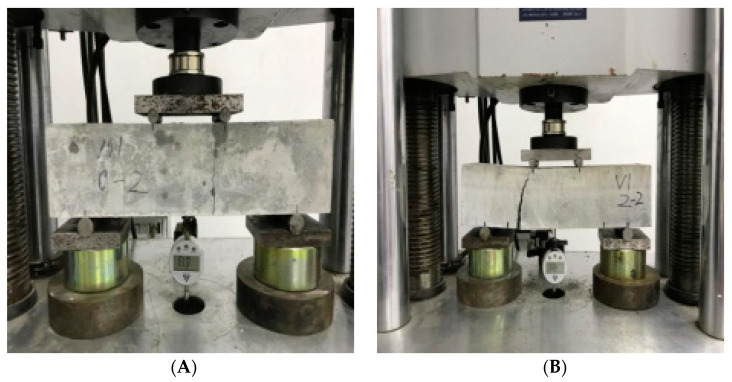
Data acquisition of steel fiber reinforced concrete crack. (**A**) Initiative crack image; (**B**) biggest crack image.

Different loads were used for pressurizing test blocks to obtain different crack sizes, in order to get diverse data. The size of the image obtained was 4032 × 3024, as shown in Figure 3. On the left (A) is the concrete test block with cracks initiating, and on the right (B) is the concrete test block with the biggest crack.

#### 3.1.3. Image Clipping

**Figure 4:**
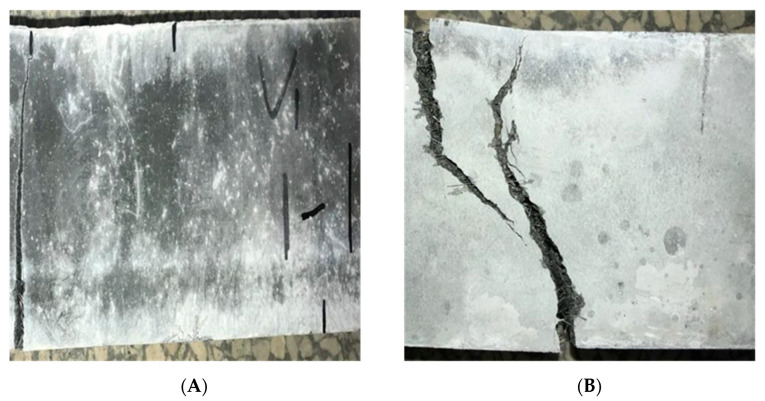
Different concrete images. (**A**) Micro crack image; (**B**) biggest crack image.

Because the resolution of the original image is large and most of it is the background area, it is meaningless for fracture recognition and time-consuming to process. Therefore, the image was cropped to the size of 512 × 512 × 3 as shown in Figure 4.

### 3.2. Image Preprocessing

**Figure 5:**
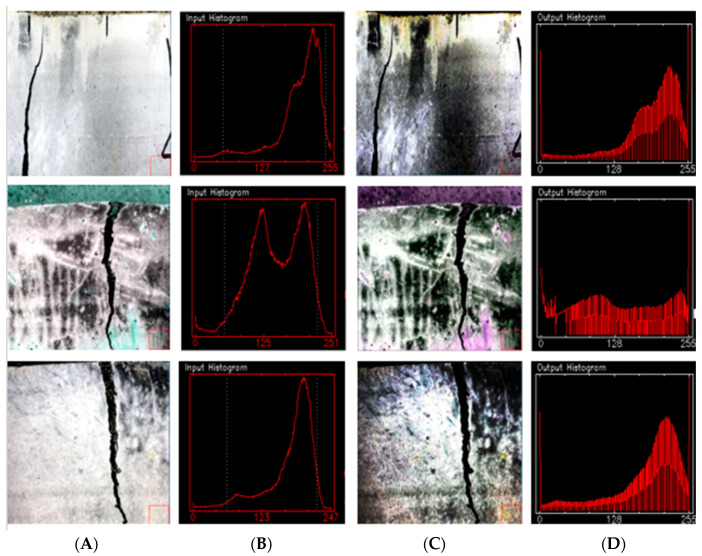
Histogram comparison. (**A**) Original image; (**B**) original histogram; (**C**) equalization images; (**D**) equalization histogram.

Histogram equalization [31] is a method to adjust image contrast. It achieves contrast adjustment by expanding commonly used brightness, and the effect is very obvious for images with dark or bright foreground and background. Since the brightness of SFRC cracks and surrounding background pixels was dark, it was necessary to perform histogram equalization on SFRC data to enhance the local characteristics of cracks. Figure 4 is the histogram comparison of steel fiber concrete crack data. The two white dash lines in the original image histogram are the pixel gray range of the corresponding image. It can be seen from Figure 5 that the gray distribution of steel fiber concrete crack data was different depending on illumination or crack area. Therefore, the balanced processing of its histogram can enhance the expression of detail information.

### 3.3. Texture Feature Extraction and Image Fusion

GLGM texture feature calculation mainly includes three parameters: statistical distance, gray level, and window size. Gray level and statistical distance determine the calculation speed and scale of GLGM. The statistical distance was set to 1 and the gray level was set to 64, referring to previous research [32].

**Figure 6:**
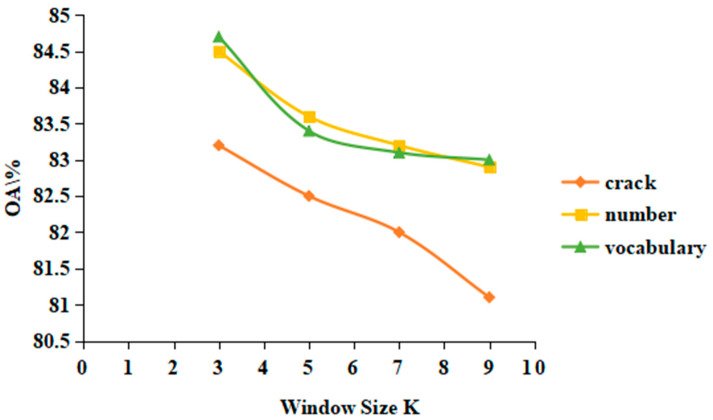
Detection accuracy comparison of different window size for GLGM.

The selection of window size is an important factor affecting texture feature extraction, which has a great impact on later target detection. Therefore, this paper compares and analyzes the selection of window size k value. Firstly, 40 training sample data were selected to extract texture features with window sizes of 3 × 3, 5 × 5, 7 × 7 and 9 × 9, and then target detection was carried out. The comparison of detection accuracy is shown in Figure 6. Through comparison, it was found that when the window size is 3 × 3, the accuracy is the highest, and the accuracy decreases with the increase of window size. Therefore, this paper sets the window size to 3 × 3. The reason for this may be related to the data pixels and sizes used in these images. If the data resolution used is high, that is, a pixel contains a small range of objects, a larger window size can be chosen.

**Figure 7:**
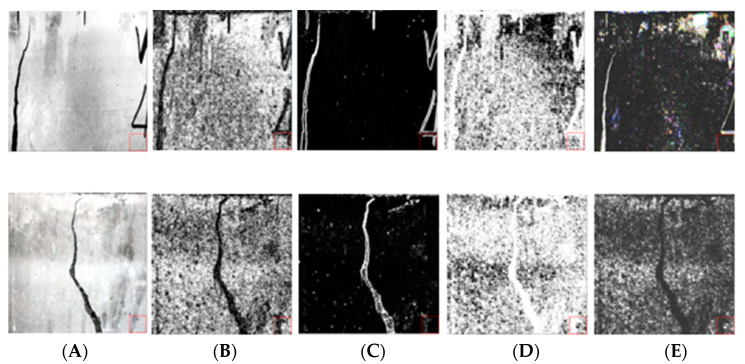
Texture feature images. (**A**) Original images; (**B**) homogeneity images; (**C**) contrast images; (**D**) entropy images; (**E**) ASN images.

**Figure 8:**
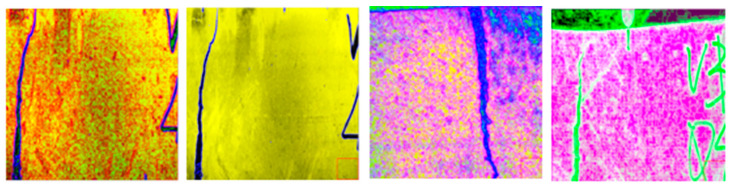
Merge texture feature images.

Using the above parameters, four texture features were extracted from the 3-band data of SFRC, and a total of 12 texture features were obtained. Figure 7 shows the four texture feature data extracted from r-band. The obtained 12 texture feature data were deeply fused with the pre-processed data to obtain 15 channel data. Figure 8 shows the data image after partial feature fusion. As shown in the figure, after fusion of data, the difference between target and background becomes clear, effectively improving the efficiency of computer recognition.

### 3.4. Sample Label Making

**Figure 9:**
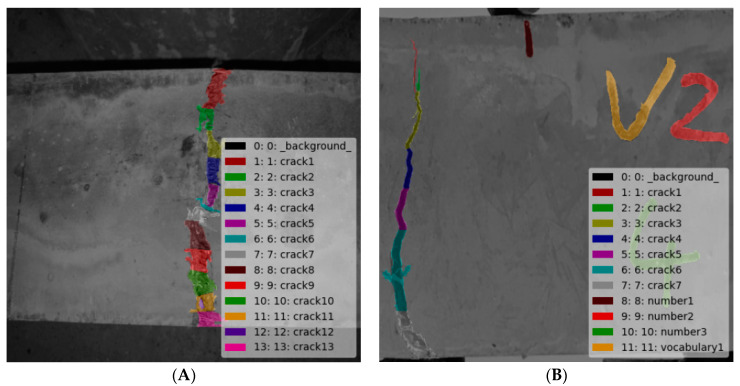
Original data label sample. (**A**) Only crack image; (**B**) three classes crack.

Because steel fiber reinforced concrete is exposed outdoors, the surrounding environment of cracks is complex, which is usually accompanied by other defects, such as leaf shielding, or corrosion which will affect crack identification. Therefore, two defects were artificially added in the production of steel fiber reinforced concrete: one is number and the other is vocabulary, which are used to simulate the external environment of concrete, so as to increase the difficulty of deep learning model training and determine the effectiveness of target recognition. Before target detection model training, it was necessary to make data labels for three types of defects. In this paper, labeling software was used to label the image. Figure 9 shows the image after making labels in the original image data.

## 4. Discussion

### 4.1. Parameter Setting

The computer configuration used for model training was I7-9800X CPU processor and NVIDIA GeForce RTX 2080 Ti graphics card. During the training of the deep learning model, 90% of the data samples were trained and the 90% data sample verification set was verified. The model of coco data set in deep learning was used as initialization parameter for training, with a learning rate of 10^−5^ and 200 iterations. By adjusting model parameters through continuous model training, the final steel fiber concrete crack detection model was obtained and saved, and the test data was tested with this model. In this paper, the best prediction result was acquired after approximately 200 training times. With the increase of training times, the detection effect of predicted sample data decreased, which may be due to the over-fitting phenomenon of the training model with the increase of training times.

### 4.2. Analysis of Experimental Results

Here, we discuss the experimental results from five aspects, including target detection accuracy analysis, detection accuracy and reliability, comparative analysis of target results, efficiency evaluation, and error analysis.

#### 4.2.1. Target Detection Accuracy Analysis

**Figure 10:**
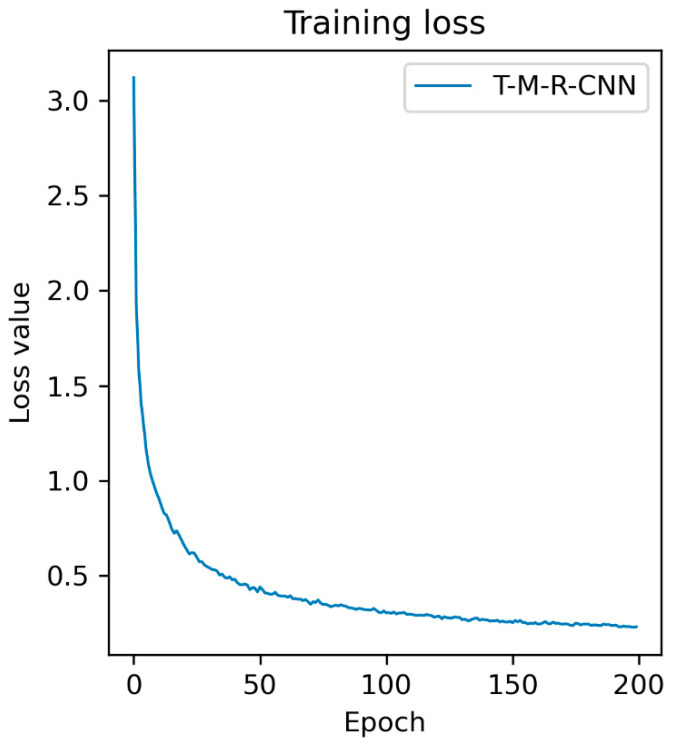
Training loss of T-M-RCNN model.

The training loss of T-M-RCNN model is shown in Figure 10. From Figure 10, it can be seen that the training loss of this model did not change much after 150 iterations. The training loss was stable at approximately 0.25. Average Precision (AP) and mean Average accuracy Precision (mAP) of target detection were used to evaluate the target detection accuracy. The specific target detection accuracy statistics are shown in Table 1. By comparing the target detection R-CNN using original images with the target detection E-R-CNN using pre-processed images, it can be found that the target detection accuracy of R-CNN was relatively low in the three kinds of defects. In terms of crack detection accuracy, the detection accuracy of E-R-CNN was 4.27% higher than that of R-CNN. Therefore, the pre-processed data can improve feature extraction in the deep learning model and thus the accuracy of target detection.

By comparing the proposed method with the target detection methods without data fusion (R-CNN and E-R-CNN), it can be found that the target detection accuracy of T-R-CNN was the best among the three defects, and the crack detection accuracy was 91.06%, 4.58% and 0.31% higher than R-CNN and E-R-CNN, respectively. The detection accuracy of Number was 89.33%, which was 1.97% and 1.38% higher than R-CNN and E-R-CNN, respectively. The detection accuracy of Vocabulary was 93.54%, which was 5.40% and 3.40% higher than R-CNN and E-R-CNN, respectively. The test results show that the proposed target detection framework had good target detection effect relative to the other models. It also had higher detection accuracy even with less training data compared with Ding et al. [24] who used 1200 crack image data to train a model with model accuracy mAP of 90.44%. It can be seen that texture features are not redundant in the process of depth features extracted by a deep learning model, and can be further abstracted to improve detection accuracy.

#### 4.2.2. Comparison of Detection Accuracy and Reliability

**Figure 11:**
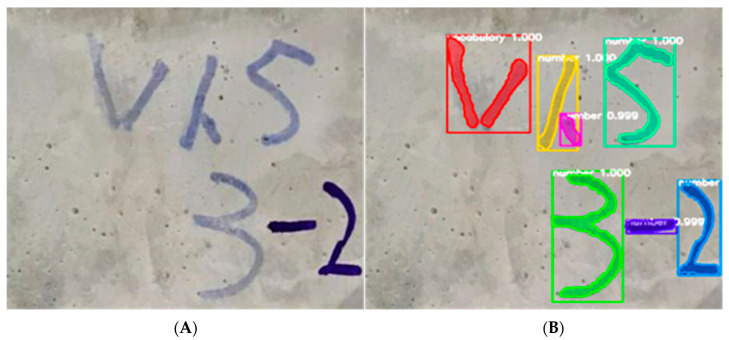
Detection result. (**A**) Original image; (**B**) target detection results, including three parts: bounding box, mask, and confidence.

Figure 11 shows the target detection results. The white numbers in the figure represent the probability that the deep learning model is considered to belong to a certain class, namely the confidence degree, which can directly measure the reliability of target detection. The higher the confidence probability value, the higher the detection reliability of the pixel. It can be seen from the figure that the confidence of most targets reached 1, and the probability of confidence of some targets also reached 0.999, indicating that the target detection effect was relatively ideal.

#### 4.2.3. Comparative Analysis of Target Results

**Figure 12:**
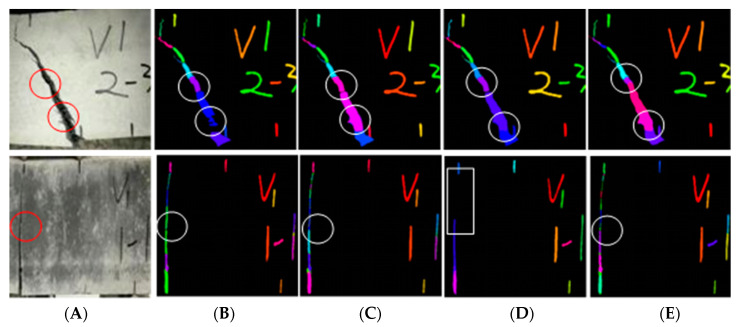
Comparison of detection targets. (**A**) Original image; (**B**) R-CNN; (**C**) E-R-CNN; (**D**) T1-R-CNN; (**E**) T-R-CNN.

Figure 11 shows some target detection results and their accuracy diagrams, where A is the original image and B-E are different detection methods of R-CNN, E-R-CNN, T1-R-CNN, and T-R-CNN, respectively. As shown in Figure 12, some obvious cracks in method B were not detected, while the detection results of methods C and D were slightly improved compared with method A, but some target information was not yet detected. The cracks detected by method E from the naked eye were not much different from the real image, which improves the target detection accuracy. In addition, it can be seen from Figure 12D that target detection accuracy of T^1^-R-CNN method, which integrates four texture feature data of R-band for deep learning, was lower than other methods. Therefore, deep learning based on texture features should be analyzed on a case-by-case basis. Although the fusion of texture features can improve the detection accuracy, it is necessary to conditionally select the original data and texture features.

#### 4.2.4. Efficiency Evaluation

When training the deep learning model, each kind of deep learning training takes the same time, indicating that the input sample does not affect the training time of the model after increasing the number of channels. The time required to train each image was about 13.24 s. A total of 90 pieces of data were used for training. The time required for each iteration was about 20 min. For 200 iterations, it took about 4000 min to complete the training. The existing deep learning model method needs at least hundreds of training data points. For example, Cha et al. [18] used 40,000 concrete crack images to construct a deep convolution network. Then it takes at least dozens of days to complete the training, consuming a longer time than the method in this paper. Table 2 compares the training time of this paper with a common number of samples used in other researches.

#### 4.2.5. Error Analysis

**Figure 13:**
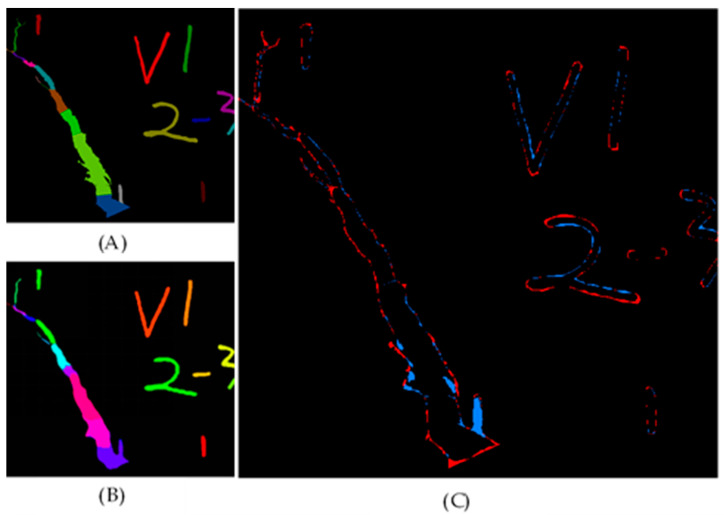
Comparison between real target detection and predicted target detection. (**A**) Real target image; (**B**) predicted target image; (**C**) compared target image.

The real target and the target image obtained by the prediction model were compared and analyzed, and the undetected target was extracted. Figure 13 shows the detection diagram of real target and predicted target, in which the red pixels represent undetected targets and the blue pixels represent incorrectly detected targets. We found that most of the incorrectly detected targets were edge parts of the target, and the main reason was that the edge part was in the transition zone between the background and the target, so the features of this part were not clear. Therefore, how to improve the detection accuracy of this part is an urgent problem to be solved in the next step.

## 5. Conclusions

This paper proposes a target detection framework combining texture features with deep learning model. Since the effect of texture feature extraction is closely related to the size of the sliding window, this paper firstly conducted a precision analysis experiment on the selection of the size K value of the sliding window to select the best sliding window. Secondly, the proposed target detection framework was tested and analyzed. The target detection framework was compared with the target detection framework without texture fusion, and the results show that the target detection accuracy of the combination of texture features and deep learning model is higher than that of traditional detection methods. Finally, the test results show that the proposed target detection framework still has good target detection effect in the case of less training data, while greatly reducing the training time and improving the training efficiency. Compared with the existing deep learning methods, the model training duration is greatly reduced, the number of samples required is correspondingly reduced, and the detection accuracy is improved to some extent.

## Figures and Tables

**Table 1:** Comparison of detection accuracy.

Method	Average Precision (AP)/%			Mean Average Accuracy (mAP)/%
	Crack	Number	Vocabulary	
M-R-CNN	86.48	87.38	88.14	87.64
E-M-R-CNN	90.75	87.95	90.14	89.61
T^1^-M-T-CNN	75.76	90.29	78.45	81.50
T-M-R-CNN	91.06	89.33	93.54	91.31

**Table 2:** Comparison of training time.

Number of Training Samples	Iteration Duration/Minute	The Number of Iterations	Total Training Time/Minute
90	19.86	200	3972
200	44.13	200	8826
2000	441.33	200	88,260
20,000	4413.33	200	882,600

## Data Availability

Data is contained within the article.
